# Signatures of optimal control in pairs of schooling zebrafish

**DOI:** 10.1098/rspb.2017.0224

**Published:** 2017-04-12

**Authors:** Andress Laan, Raul Gil de Sagredo, Gonzalo G. de Polavieja

**Affiliations:** Champalimaud Neuroscience Programme, Champalimaud Center for the Unknown, Lisbon, Portugal

**Keywords:** collective behaviour, interaction rules, optimal control

## Abstract

Animals moving in groups coordinate their motion to remain cohesive. A large amount of data and analysis of movement coordination has been obtained in several species, but we are lacking theoretical frameworks that can derive the form of coordination rules. Here, we examine whether optimal control theory can predict the rules underlying social interactions from first principles. We find that a control rule which is designed to minimize the time it would take a pair of schooling fish to form a cohesively moving unit correctly predicts the characteristics of social interactions in fish. Our methodology explains why social attraction is negatively modulated by self-motion velocity and positively modulated by partner motion velocity, and how the biomechanics of fish swimming can shape the form of social forces. Crucially, the values of all parameters in our model can be estimated from independent experiments that need not relate to measurement of social interactions. We test our theory by showing a good match with experimentally observed social interaction rules in zebrafish. In addition to providing a theoretical rationale for observed decision rules, we suggest that this framework opens new questions about tuning problems and learnability of collective behaviours.

## 1. Introduction

Autonomous systems in general, and animals in particular, often benefit from coordinating their behaviour with other similar agents [1,2]. In suitable conditions, highly complex and coordinated multi-agent behavioural displays may emerge through local social interactions. Many decades of research has uncovered the functional rationale underlying the various forms of collective behaviours, which can range from increased protection from predation and risk dilution [3] to optimal information use and accelerated learning [4].

An improved understanding of the functional roles of collective behaviour has opened the way to theoretical analyses that derive the rules of collective behaviour from first principles. One example originates from the study of decision-making in collectives. While early models were mostly phenomenological [5] (but see Condorcet theorem [6] and information cascades models [7]), more recently, Bayesian estimation theory has provided a gateway for predicting biologically observed decision rules through a treatment grounded in probability theory [8,9].

In the study of collective motion, phenomenological models still dominate over normative models. Many heuristic and data-driven models have provided highly successful descriptions of varied systems like fish schooling, pedestrian traffic flows, avian aerial displays and ant trail construction [1]. When attempts have been made to theoretically predict the finer details of social interactions through, for example, evolutionary computing [10], the underlying functional form of animal interactions has been based on heuristic assumptions or measurements [11–14] rather than theoretical considerations (but see [15]).

While phenomenological models use mathematical equations to fully specify agent behaviour, these equations are arrived at primarily through the aid of intuition or by fitting equations of motion to data. For new examples, the modeller is given many opportunities to change the form of the equations any time an application shifts to a new species or domain. Normative models are different. Rather than specifying the governing equations directly, the theorist specifies an underlying logical framework from which equations can be derived for particular examples. When successful, normative models usually achieve greater generality, a better grounding of the models in experimental measurements and allow explicit connections to be made between mechanistic and functional explanations. An *ab initio* prediction of rules governing social interactions has remained an unsolved challenge.

Here we demonstrate that control theory makes predictions about interaction rules for pairs of schooling fish which are qualitatively consistent with experimentally measured social forces in zebrafish. In our control theory framework, we set out to solve the following problem. The sensory system of a fish has access to its own velocity, the velocity of its partner fish and their mutual distance. The locomotor system, on the other hand, can specify the acceleration produced by the muscles of the fish. What relationship between the sensory inputs of the fish and its locomotor output would enable the schooling fish to maximize the cohesion of the group?

We developed a control-theoretic treatment of fish schooling and we compared theoretical predictions against experiments in an effort to test the applicability of our framework on the domain of collective behaviour. While our treatment is grounded in the known biology of the fish locomotor system, we do make the simplifying assumption that fish move in a quasi-one-dimensional environment. This is done because in our set-up, zebrafish spend 80% of their time swimming parallel to the walls along approximately one-dimensional tracks and our access to two-dimensional data is thus more limited. In addition, to the best of our knowledge, the biomechanical constraints of turning in fish are less well understood, which has limited our ability to create a two-dimensional theory of turning.

## 2. Material and methods

### (a) Experimental set-up

Ten-month-old zebrafish from the five-dimensional strain were kept in a tank with 40 individuals (equal numbers of male and female) in a 10 L : 14 D cycle from which pairs of animals were randomly sampled for schooling experiments. We recorded 13 pairs of zebrafish swimming in a 35 × 35 cm tank with water depth of 5 cm for 30 min. The fish were recorded at 30 fps and the resulting videos were tracked using idTracker [16] giving as output 26 individual trajectories. The trajectories were first smoothed with a 170 ms moving average filter (or 5 out of 30 frames per second). The *x*-components of velocity and acceleration at time *t*_*i*_ were calculated as *v*_*x*_(*t*_*i*_) = *x*(*t*_*i*_) − *x*(*t*_*i*−1_) and *a*_*x*_(*t*_*i*_) = *v*_*x*_(*t*_*i*+1_) − *v*_*x*_(*t*_*i*_), respectively, where *t*_*i*_ = *i*/30, with *i* the frame number and *t*_*i*_ the acquisition time of that frame in seconds.

### (b) Kinematics

For analysis of viscous drag, the local maxima and minima were extracted for each time series of the velocity waveform. The descending phases of the velocity trajectory were localized and each local descending region was fitted with an equation 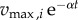, where 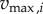 was the amplitude of velocity at the *i*th local maxima. The parameter *α* was shared for all local descending regions and all fish. The value of *α* was chosen such that the mean absolute deviation between the fit and the observed trajectories was minimized. For quadratic drag, the same procedure was used except for the equation which was written as 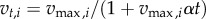.

### (c) Predictability of acceleration

The amount of unexplained linear variation was calculated as 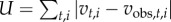, where *v*_*t*,*i*_ was the predicted velocity at time *t* of descent episode *i* and *v*_obs,*t*,*i*_ was the observed velocity. The total linear variation was calculated as 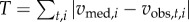, where *v*_med,*i*_ was the median velocity of the *i*th decay waveform. The total fraction of explained linear variation is *f* = (*T* − *U*)/*T*.

In order to analyse the predictability of acceleration using social information, we used an artificial neural network with eight input units representing the *x*- and *y*-coordinates and the *x*- and *y*-components of the velocity of the focal and partner fish at time *t*. All input signals were normalized to unit variance and had zero mean. The output signal was the *x*- and *y*-components of the acceleration at time *t* + 1. We used one hidden layer with 40 neurons and fitted the net individually for each fish after splitting its data randomly into a training set (70% of data), validation set (15%) and test set (15%). Nets were fitted with Levenberg–Marquard optimization using MATLAB neural networks toolbox. For predicting acceleration at time *t* + 1 from its value at time *t*, ordinary linear regression was used.

### (d) Empirical forcemaps

The forcemaps were extracted by analysing how the component of acceleration of the focal fish that was parallel to focal fish velocity depends the position of the other fish in a focal fish centric coordinate system (procedure as in [11]). The distance of the other fish was also measured using the position vector component that was parallel to the velocity of the focal fish. The velocity of the other fish was similarly projected onto the velocity of the focal fish and the amplitude of the projection was used for analysis.

The social forcemaps might potentially suffer from contaminating data from periods of non-social interaction. In order to correct for this potential bias, we analysed the expected number of instances in which a temporally shuffled dataset would generate observations within the variable range of our forcemaps. We found this number to be less than 25% of total observations in our experimental forcemaps. We corrected the amplitude of social forces by multiplying the observed forces by the number 1/(1 − *k*), where *k* is the total number of observations found in a shuffled forcemap divided by the total number of observations in the non-shuffled forcemap.

## 3. Results

### (a) Characteristics of zebrafish swimming

**Figure 1.**
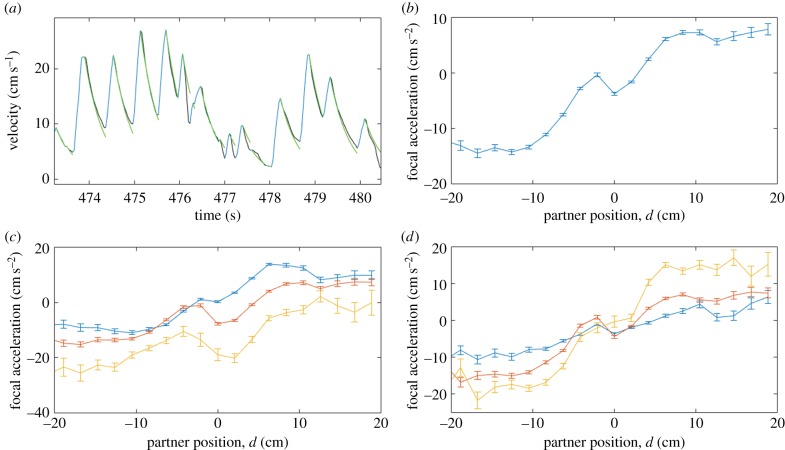
Analysis of zebrafish swimming. (*a*) Example of magnitude of velocity over time for one fish showing acceleration (blue) and deceleration phases (black). Also shown are exponential decay fits to decelerating phases (green). (*b*) One-dimensional social force as a function of the relative distance between focal fish and partner fish. (*c*) Modulation of social forces by focal fish velocity. Focal fish velocity ranges: 0–10.5 (blue), 10.5–21 (red) and 21–32.5 (yellow). (*d*) Modulation of social forces by partner fish velocity. Partner fish velocity ranges: 0–10.5 (blue), 10.5–21 (red) and 21–32.5 (yellow). *n* = 13. Bars are s.e.m.

A control-theoretic treatment of fish locomotion begins with a characterization of the motor output of the fish. We first obtained a description of the forces at play in zebrafish swimming in one dimension. As in many fish species, zebrafish swimming is a highly structured temporal process [17]. Active periods of near-maximal acceleration alternate with periods of passive gliding where deceleration occurs due to viscous drag. This process generates a saw-tooth-like evolution of the velocity profile (figure 1*a*). We used the deceleration phase of the locomotor cycle in zebrafish to characterize the drag dynamics. We found that simple exponential decay accounted for 62% of the linear variations in speed. This performance measure was calculated across all decay waveforms for all fish using only a single free parameter shared across all traces, thus characterizing the average viscous drag on the body of a zebrafish (see ‘Kinematics’). This is consistent with viscous drag forces proportional to velocity, with acceleration3.1
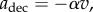
and *α* = 2.7 s^−1^ in our case. A drag force quadratic in velocity gives a worse fit accounting for 50% of variation. The rising phase of the velocity profile is well described by an acceleration towards maximal velocity 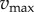 as3.2
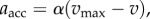
with 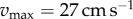. This value is consistent with the observed velocities, with 95% of them below this value. The few instances of observed velocities above 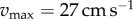 might be accounted for by rare periods of anaerobic acceleration.

In experiments using pairs of zebrafish, active swimming dynamics still strongly shapes swimming patterns. The acceleration of a fish at time *t* predicts 45 ± 0.76% of the linear variation in its acceleration at time *t* + 1 (see ‘Predictability of acceleration’). By contrast, a neural network provided with spatial information and the velocity vectors of both the focal fish and its partner fish at time *t*, which characterizes the social information available to a fish, can explain only 6.5 ± 0.3% of acceleration at time *t* + 1 (see ‘Predictability of acceleration’). Our results contrast with a common view of social interactions, which concentrates on the influence of social parameters such as the relative inter-individual position, heading and velocity as determinants of future movements. In our experiments, the non-social part of the locomotor cycle is a better predictor of future acceleration values than social information even in social settings. The strong influence of non-social processes on behaviour is consistent with previous reports [18–20].

To extract the characteristics of social interactions, we used the forcemap approach [10,11]. It consists of computing the dependency of the mean social force (focal fish mean acceleration) on inter-animal distance and velocity of focal and partner. In our set-up, zebrafish spend 80% of their time swimming parallel to the walls along approximately one-dimensional tracks. We restricted our analysis to periods where zebrafish velocity vectors were highly parallel to each other, with differences in heading angles less than 45°, the distance between fish in the direction perpendicular to focal fish velocity vector less than 7 cm and the distance of fish in the parallel direction less than 20 cm (a total of 30% of all data were thus used for analysis).

From these approximately one-dimensional tracks, we measured how acceleration of a focal fish depends on its distance to its partner, the speed of the partner and the speed of the focal fish. A partner fish located in front (behind) of the focal fish generates a strong positive (negative) acceleration response in the focal fish (figure 1*b*). The velocity of the focal fish modulates the acceleration of the focal fish in a negative fashion: increasing focal fish velocity causes increased deceleration (figure 1*c*). This is consistent with passive viscous forces both reducing the maximal acceleration attainable to a fast-moving fish as well as increased viscous deceleration experienced during the gliding phase. By contrast, increasing partner fish velocity appears to promote increased acceleration of the focal fish, particularly when the partner fish is located in front of the focal individual (figure 1*d*).

### (b) Social interactions from optimal controller

A theoretical treatment of group swimming must also specify the objective of the fish. This objective must be expressible in terms of observed variables and should capture the biological intuition that schooling fish are trying to move in a cohesive manner. We define a pair of fish as moving with maximal cohesion if both individuals are moving with identical velocities and are positioned as close to each other as physically possible. We next derived an approximately optimal controller which tries to maintain maximal cohesion in order to compare whether such a controller might explain the observed characteristics of zebrafish social interactions.

Our controller takes as input the relative position of the partner fish, as well as velocities of both the focal and partner fish, and it outputs a dynamic decision which specifies whether the focal fish should glide or accelerate. The controller is designed to optimize the time it takes to bring two fish into a maximally cohesive state from any initial state. The minimum time objective was chosen partly because it might minimize predation risk by allowing a dispersed group of fish to arrive at a maximally cohesive and presumably safest group configuration in the minimum amount of time. A second reason behind our choice of cost function is mathematical. Minimum time controllers are easy to optimize. It remains possible that other cost functions such as minimization of inter-fish distance over time will also produce behaviours consistent with data. The exploration of alternative cost functions is left as future work.

The Pontryagin minimum principle specifies the bang–bang controller as the optimal controller for our scenario [17,21] (while this is a standard result, we have also added the sketch of a proof for our particular problem to the electronic supplementary material). A bang–bang controller prescribes that if an agent should decide to accelerate, it should do so with maximal intensity. Deceleration decisions should likewise use only passive gliding to attain maximal possible deceleration.

The decision-making of a zebrafish is divided into a simple classification problem: accelerate maximally or glide. From these arguments, we obtain that the amplitude of deceleration should be equal to *a*_F_ = *a*_dec_ = −*αv*_F_ and the amplitude of acceleration should be given by 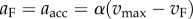, where *v*_F_ is the velocity of the focal individual and 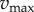 is the maximal possible speed obtainable through aerobic swimming.

The decision to glide or accelerate can then be formalized as a three-dimensional decision problem with inputs *v*_F_ (focal fish velocity), *v*_P_ (partner fish velocity) and *d* = *x*_partner_ − *x*_focal_ (inter-animal distance). We derive the location of the decision boundary from the following standard argument: if a zebrafish is located on the decision boundary and the partner fish is expected to maintain a constant velocity *v*_P_ (see ‘Discussion’ about possible caveats associated with this assumption), then passive gliding will eventually bring the focal fish to a maximally cohesive state (same point in space and same velocity). In the electronic supplementary material, we derive the equation describing this decision surface as3.3
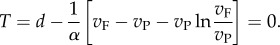


The resulting decision rule then corresponds to fish accelerating, *a*_F_ = *a*_acc_, at one side of the decision surface, *T* > 0, or decelerating, *a*_F_ = *a*_dec_, at the other side of the decision surface, *T* < 0. Additionally, when it is the focal fish that is in front of the partner, *d* < 0, it glides until *d* > 0. In summary, the rule is completely characterized by the following equations describing how the focal agent should accelerate:3.4
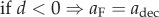
3.5
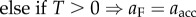
3.6
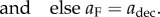


**Figure 2.**
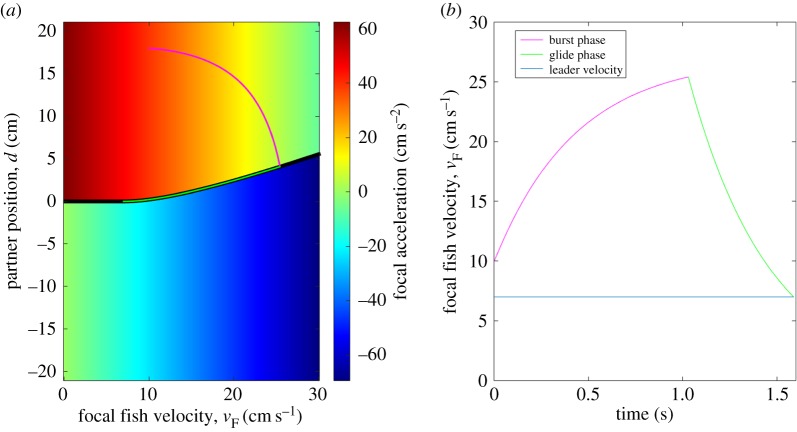
State space depiction of the optimal control rule. (*a*) Colourmap depicting how the acceleration of the focal fish changes as a function of the velocity of the focal fish and the inter-agent distance (position). For this figure, the velocity of the partner fish is held constant at 7 cm s^−1^. The black line depicts the decision boundary. Above the boundary, fish engage in burst acceleration, below the boundary in gliding. An example trajectory of a focal fish is depicted in the state space with burst (purple) and gliding (green) phases. (*b*) Time-evolution of the velocity profile of the focal fish for the state space trajectory depicted in (*a*). Blue horizontal line: velocity of partner fish.

Our decision rule is fundamentally a three-dimensional process, but it can be visualized as a series of two-dimensional sections. In figure 2*a*, we depict one such cross section, where we have fixed the value of the partner fish velocity in order to illustrate how changes in focal fish velocity and the inter-animal distance influence acceleration decisions. The decision boundary is visualized as a black line, which separates the acceleration zone from the deceleration zone. As can be seen from the figure, increasing focal fish velocity increases deceleration, as is apparent from the progressively deeper blue tones as we move from the left to the right on the graph.

One advantage of our two-dimensional visualization is that focal fish behaviour can be visualized as trajectories in the state space graph. In figure 2*b*, we have depicted one such example trajectory in the state space. During the burst phase (purple), the focal individual increases its velocity and decreases its distance relative to the partner fish until it reaches the decision boundary. After reaching the decision boundary, the focal individual switches to gliding mode (green) and moves along the decision boundary until its position and velocity match with its partner. The resulting evolution of the velocity of the focal fish is depicted in figure 2*b*, with a saw-tooth-like velocity waveform from the switch from bursting to gliding.

**Figure 3.**
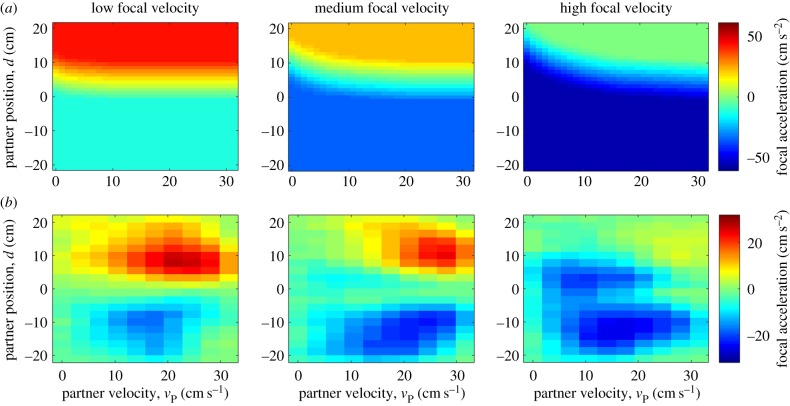
Comparison of optimal controller and zebrafish social forces. (*a*) Theoretical predictions of three-dimensional decision maps. On each colourmap, we plot for a restricted range of values of focal fish velocity how acceleration of zebrafish varies as a function of partner fish velocity and the relative distance between focal and partner fish. Velocity ranges from left to right were 0–10.5, 10.5–21 and 21–32.5 (cm s^−1^). For the calculations, we have also added noise to the sensory system of the focal fish which causes an imprecise estimate of the location of the partner fish (see the electronic supplementary material, figure S1 for noiseless predictions). (*b*) Same as (*a*) but for zebrafish experiments (*n* = 13 pairs).

We present an alternative depiction of the structure of our optimal controller, where we fix to a limited range the velocity values of the focal fish in order to see the effects of varying partner fish velocity (figure 3*a*). This was modelled with sensory noise added in the estimation of interfish distance (see the electronic supplementary material, methods 5.3 for details of noise modelling and comparison with noise-free predictions). We also provide analogous plots of the experimentally measured zebrafish interactions (figure 3*b*). The optimal controller has three features that are qualitatively consistent with the measured decision maps. First, the amplitude of the deceleration and acceleration zones are negatively modulated by the focal zebrafish velocity. Second, large values of partner fish velocities activate acceleration behaviours when the partner fish is located in front of the focal fish. Third, the transition zone between the acceleration and deceleration zones has a negative slope in the *d*–*v*_partner_ axis.

Overall, both the biological controller and the optimal controller have a simple intuitive structure. If the partner fish is located in front of the focal fish and moving faster than the focal fish, then the focal fish should accelerate. This behaviour leads to an increase in its speed and will allow it to catch up with the partner fish. The acceleration should continue until the focal fish reaches a sufficient speed and position such that a switch to passive gliding is expected to bring it into a maximally cohesive state with its partner. If the speed of the focal fish is much greater than the speed of the partner fish, then the focal fish should always glide. Gliding causes the passive drag of water to bring its velocity closer to the velocity of its partner and the inter-individual distance will simultaneously shrink as well, since the greater speed of the focal fish relative to the partner will cause the focal fish to approach the partner. Finally, if the focal fish is located in front of the partner fish, then due to the blind angle, a precise estimation of the position and velocity of the partner fish is not possible and the best decision is to glide passively until the partner fish overtakes the position of the focal fish.

**Figure 4.**
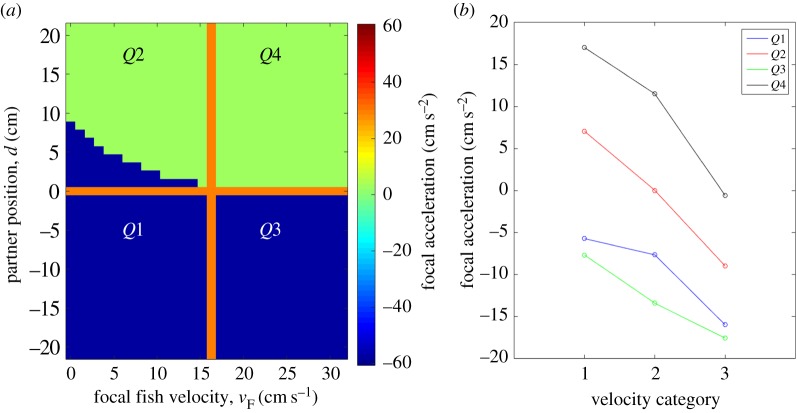
Analysis of quadrants of experimental control surface. The two-dimensional forcemaps were divided into four quadrants and the average acceleration was computed for each for the three groupings of focal fish velocity (*n* = 13 pairs): group 1, 0–10.5; group 2, 10.5–21; group 3, 21–32.5 (cm s^−1^). Consistent with theory, all quadrants are negatively modulated by focal fish velocity. The relative ranking of the acceleration amplitudes is also largely consistent with theory, which predicts that *Q*4 > *Q*2 > *Q*3 ≈ *Q*1.

This qualitative consistency can be seen in figure 4. We have divided the forcemaps into four quadrants (figure 4*a*) and plotted how the average acceleration in each quadrant varies with focal fish velocity (figure 4*b*). Consistent with theory, acceleration in all four quadrants is negatively modulated by focal fish velocity. The relative ranking of the intensity of acceleration in all quadrants is also qualitatively consistent with theory. Quadrants *Q*1 and *Q*3 have the lowest values of acceleration, because they correspond to situations where the partner is located behind the focal fish and the focal fish is always decelerating. Quadrants *Q*2 and *Q*4 partially overlap with acceleration regions so they show higher acceleration values. Because quadrant *Q*4 corresponds to higher values of the partner fish velocity, it has higher average acceleration values than quadrant *Q*2.

While the controller map is qualitatively consistent with observed zebrafish behaviour, there are quantitative differences. Notably, the amplitudes of the acceleration and deceleration zones are approximately 40% weaker than predicted by theory and the transitions between acceleration and deceleration zones are less abrupt. The sources of this discrepancy are further addressed in the next section, which details a simulation approach to study contributions of non-social behaviour to the empirical forcemaps.

One prominent feature in both the optimal controller and biologically measured controllers is velocity-dependent modulation of social interactions. In the electronic supplementary material, we show mathematically that velocity-dependent modulation of social interactions is a necessary characteristic of fish social interactions given other features known about fish swimming control systems such as the relative dominance of frontal acceleration zones over caudal deceleration zones as well as the use of egocentric representations of decision rules. Control theoretic analysis is thus more generally able to successfully explain many qualitative features of social interactions in fish collectives.

### (c) Closing the loop through simulations

We next attempted to close the loop by simulating fish following the optimal control rule to see if we could find a match between the most relevant summary statistics of fish locomotion in experiments and simulation.

Closing the loop required making some assumptions about the non-social behaviour of fish. There is a leader fish, who moves non-socially (it does not respond to the movements of the follower) in a burst and glide swimming pattern. We tested for leadership in zebrafish by examining velocity vector cross-correlations, which showed that zebrafish pairs exhibit both asymmetric and alternating leadership patterns (electronic supplementary material, figure S2). The follower fish obeyed our control rule except for non-social periods where it exhibited burst and glide behaviour (see the electronic supplementary material). The probability of the follower being in a non-social state was 0.2 and the duration of a non-social episode was 15 s on average (similar to the 5–15 s duration of shoal cohesion oscillations in zebrafish [18]).

**Figure 5.**
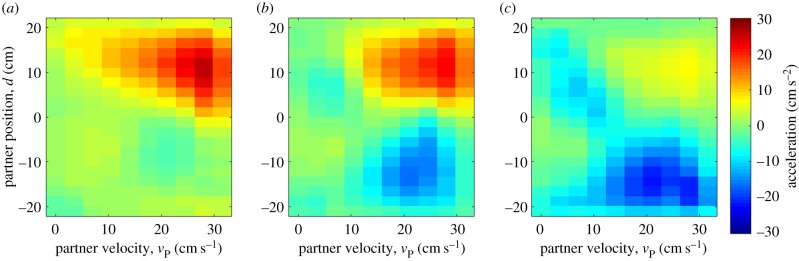
Forcemaps extracted from simulated trajectories. (*a*–*c*) Same as for figure 3 but for simulated fish trajectories (see main text for details) instead of ideal controller. Velocity ranges were (*a*) 0–10.5, (*b*) 10.5–21 and (*c*) 21–32.5 (cm s^−1^).

To calculate the forcemaps, we summed the occupancies and accelerations of both the leader and the follower fish in each bin, and then calculated the averaged forcemaps by dividing the net acceleration with the net occupancy. This is important because zebrafish leadership can switch unpredictably during the recording session and our experimental forcemaps contain contributions from both the leader and the follower fish. We subsequently extracted empirical forcemaps from simulated trajectories and found them to have a good qualitative match with experimental measurements (figure 5; cf. figure 3).

We note the following key features: even though our control rule generated peak acceleration values of 60 cm s^−2^, the simulated forcemaps have peak acceleration amplitudes of 25 cm s^−2^, which is much closer to the experimentally measured peak values of 26 cm s^−2^. It appears that non-social behaviour dilutes the contribution of the control rule both in simulation and in experiments. A second feature of the simulations which is qualitatively consistent with the experimental forcemaps is the non-uniform appearance of acceleration and deceleration zones. For example, in the medium- and low-velocity maps in both experiments and simulations, regions of peak acceleration are concentrated in the lower part of quadrant 4. Such peaks are absent from the theoretical approximations and again suggest the importance of the influence of non-social periods of activity on the maps. Our simulations also closely reproduced key summary statistics of fish velocity (simulations: 13.5 ± 5.8 cm s^−1^ and 12 ± 6.5 cm s^−1^).

Even in our simulations a certain mismatch remains with experiments. First, the simulated deceleration zones tend to have smaller amplitudes. This may be caused by an overly simplistic model of non-social fish behaviour, which lacks known features like homebase behaviour [22], sporadic aggression [16], and switching between fearful and more pacific internal states [23]. Second, the median interfish distance in our simulations was 4.6 cm while in experiments this measure was 5.6 cm. This is easily explained because in experiments fish often spend extended periods exploring the arena. Overall, our simulations reproduced the most salient experimental features of zebrafish schooling.

## 4. Discussion

Early phenomenological descriptions of fish schooling used self-propelled particle models where agents kept a constant velocity [1,13]. Experimental measurements of schooling recently found that changes in the magnitude of velocity were integral to the process of schooling [11,12]. The same studies also first described velocity-dependent modulation of social forces although the functional reason behind this modulation was not discussed. Our theoretical framework provides a strong rationale behind this phenomena. Velocity-dependent modulation of interactions is a necessary precondition for stable schooling in fish species where acceleration and deceleration zone amplitudes have a rostro-caudal asymmetry and the frontal acceleration zone has a stronger amplitude than the caudal deceleration zone. A more precise coordination of velocity-dependent forces can even be used to construct a near-optimal controller, and signatures of such a controller appear present in the experimental data for zebrafish.

The results of our study suggest many new avenues of experimental research. One exciting approach concerns inter-species comparison of schooling. Different fish species exhibit different locomotor dynamics and it will be important to test the effects of altered locomotion on the characteristics of forcemaps. The characteristics of forcemaps are also influenced by the behaviour of fish during non-social episodes. It would be interesting to apply our approach to fish species where leadership is less dynamic and more asymmetric than in zebrafish. In such species, where leaders and followers are easier to separate, the leader and follower maps might look substantially different [24]. Comparing the predictions of theory with experiments in these species represents a further important test of our theory.

The presence of signatures of optimal control in simple situations where pairs of zebrafish interact in a quasi-one-dimensional geometry raises multiple questions. One is how the framework extends to two- and three-dimensional scenarios and to larger group sizes. Another is how fish estimate the variables needed for real-time computation of the decision boundary from their retinal image. A third is how these variables are re-estimated during development, as changes in fish body shape modify viscous drag.

We believe that a joint solution to these problems can be found through the methodology of deep multi-agent reinforcement learning [25,26]. Multi-agent reinforcement learning can learn to solve complex control problems in situations where analytical methods of traditional control theory are no longer tractable. Extensions of our model to multi-agent three-dimensional scenarios would be one such example situation, although this extension will also require modifications to our cost function, which is currently only stated for the case of pairwise interactions. Deep reinforcement learning also provides a solution to the sensory estimation problem, because it can learn useful policies directly from high-dimensional data such as retinal activity patterns. As deep reinforcement learning is fundamentally an adaptive method which responds to changes in the environment or the body of the zebrafish, it also provides a natural solution to the problem of tuning the decision boundary [15,27] over developmental time scales.

Multi-agent deep reinforcement learning is not only conceptually attractive [28], but also inherently biologically plausible, because the neural apparatus needed to implement its computations is present in vertebrate brains [29]. One potential challenge to the use of deep learning in schooling is the large hunger for data often needed to train such systems. Our preliminary computational experiments indicate that this is not a problem for the case of schooling in pairs, because an artificial neural network composed of just 200 neurons is capable of learning competent schooling policies using data gathered in under 30 min of real-time schooling behaviour.

The present work has outlined how control theory can provide a simple mathematical description of zebrafish schooling in pairs. We believe that the presence of simple signatures of optimal control in relatively simple scenarios is not accidental but a sign of a more complex controller, which (among many other problems) is also able to find near-optimal ways to coordinate collective behaviour in simple scenarios. An important future extension of this work would explore whether methods such as deep reinforcement learning could provide a unified description of the diversity of social behaviours seen in different species and in different environments.

## Supplementary Material

Supplementary Information

## Data accessibility

Data are available from Dryad Digital Repository [30].
