# Supplementary material for: Signatures of optimal control in pairs of schooling zebrafish
Source: Proc Biol Sci. 2017 Apr 12;284(1852):20170224. doi: 10.1098/rspb.2017.0224 (PMC5394674; doi:10.1098/rspb.2017.0224)
Supplement: Supplementary Information [file rspb20170224supp1.pdf]

Signatures of optimal control in pairs of schooling zebrafish: Electronic Supplementary Material

February 23, 2017

Andres Laan[1,*], Raul Gil de Sagredo [1] & Gonzalo G. de Polavieja[1,*]

[1] Champalimaud Neuroscience Programme, Champalimaud Center for the Unknown, Lisbon, Portugal

* andres.laan@neuro.fchampalimaud.org
* gonzalo.polavieja@neuro.fchampalimaud.org

# Derivation of the equation for the decision boundary in the case of linear drag.

The equation of motion for the velocity of a gliding fish can be written as:

$$\frac{dv}{dt} = -\alpha v, \tag{1}$$

which, when integrated gives

$$v_t = v_0 e^{-\alpha t}, \tag{2}$$

where α is the coefficient of viscosity, v_t is velocity at time t and v_0 gives the velocity at time 0. We consider that a focal fish and a partner fish in front, and we will write focal and partner velocities as V_F and V_L to remind us of this leadership-followership relationship. If we assume that the leader fish initially has constant velocity v_L and the follower fish travels at v_F (with

$v_L < v_F$), then the follower fish can glide and its velocity becomes equal to the leader fish velocity,$V_F = v_L$ at time t_{eq} given by

$$t_{eq} = \frac{1}{\alpha} \ln \frac{v_F}{v_L}. \tag{3}$$

During this time, the follower fish travels a distance

$$s_F = \int_0^{t_{eq}} v_F e^{-\alpha t} dt = [\frac{v_F}{-\alpha} e^{-\alpha t}]_0^{t_{eq}} = \frac{v_F}{\alpha}(1 - \frac{v_L}{v_F}) = \frac{1}{\alpha}(v_F - v_L) \tag{4}$$

while the leader crosses the distance $v_L t_{eq} = \frac{v_L}{\alpha} \ln \frac{v_F}{v_L}$. This means that when the follower fish starts to glide from velocity v_F at a distance from the leader fish of

$$d = \frac{1}{\alpha}[v_F - v_L - v_L ln \frac{v_F}{v_L}] \tag{5}$$

it will achieved maximal cohesion between the states of the leader and the follower fish, which is to have $v_L = v_F$ and $d = 0$.

Derivation of the equation for the decision boundary in the case of quadratic drag.

Here we give a derivation analogous to the one in previous section but for the case of quadratic drag. The equation of motion for the velocity of a gliding fish can be written as

$$\frac{dv}{dt} = -\alpha v^2, \tag{6}$$

which, when integrated gives

$$v_t = \frac{v_0}{1 + v_0 \alpha t}, \tag{7}$$

where α is the coefficient of viscosity, v_t is velocity at time t and v_0 gives the velocity at time 0. If we assume that the leader fish initially has velocity v_L and the follower fish travels at v_F (with $v_L < v_F$), then the follower fish velocity becomes equal to the leader fish velocity at time t_{eq} given by:

$$t_{eq} = \frac{v_F - v_L}{\alpha v_F v_L} \tag{8}$$

During this time, the follower fish travels a distance:

$$s_F = \int_0^{t_{eq}} \frac{v_F}{1 + v_F \alpha t} dt = [\frac{1}{\alpha} ln(1 + v_F \alpha t)]_0^{t_{eq}} = \frac{1}{\alpha} ln \frac{v_F}{v_L} \tag{9}$$

while the leader crosses the distance $v_L t_{eq} = \frac{v_F - v_L}{\alpha v_F}$. This means that when the follower fish starts to glide from velocity v_F at a distance from the leader fish of

$$d = \frac{1}{\alpha}[ln(\frac{v_F}{v_L}) - \frac{v_F - v_L}{v_F}] \tag{10}$$

it will achieved maximal cohesion between the states of the leader and the follower fish, which is to have $v_L = v_F$ and $d = 0$.

Proof of the bang-bang controller for our specific case

A central principle of optimality is that all perturbations to the optimal solution will result in a worse solution than the optimal one. In the case of a burst and glide pattern, we may consider all possible perturbations to the optimal solution and we must then show that they result in a worse solution than the optimal one.

Let us consider the motor output for the case of the optimal burst and glide pattern. The motor output is bounded between zero (passive gliding) and one (maximal acceleration). For any particular optimal trajectory, a fish will be in some initial state at time zero, it will accelerate until time T_1 and then glide until time T_2. Between the times zero and T_1, the motor output will be maximal. Therefore, any perturbation will need to have a motor output value equal to 1 or smaller during that period. If at least during one point, the motor output is smaller than 1, then the velocity for at least one point during the time $t < T_1$ will be smaller than the velocity was at that time for the optimal trajectory. In other words: $v_p(t) \leq v_o(t)$ for all $t \leq T_1$, where v_p is velocity of perturbed trajectory and v_o is velocity of optimal trajectory. Therefore, the distance traveled, which is the integral of the velocity, will also be smaller for the perturbed trajectory.

For any perturbed trajectory for times $t < T_1$, we have seen that both the distance traveled and the velocity will be smaller than they were for the case of the optimal trajectory. During the second period ($T_1 < t < T_2$), when compared with the optimal trajectory, the fish can only have a motor output of equal or greater intensity than it did for the case of the optimal trajectory. Now, two scenarios are possible. In the first scenario, $v_p < v_o$ also for all $T_1 < t < T_2$. In that case, the fish will travel a net distance that was shorter than for the case of the optimal trajectory and it therefore won't catch up to the partner fish at time T_2. In the second case, at one point in time (lets call this time t_c), $v_p > v_o$. If that is the case, then the distance traveled may potentially be longer than it was for the case of the optimal

trajectory and the interfish distance may become zero at some point in time that is earlier than T_2. But in this case, v_1 will remain higher than v_o for all times $t_c < t < T_2$. This is true because for all times $t_c < t < T_2$ the fish with the optimal control input had a motor output as small as was possible. Therefore, any other fish with a velocity value v_1 cannot achieve a velocity smaller than v_o during the period $t_c < t < T_2$.

Hence, we have shown that all possible perturbations to the optimal motor output will result in either an insufficiently small distance or an excessively large velocity at all times $t < T_2$ and therefore they cannot be better than the optimal trajectory.

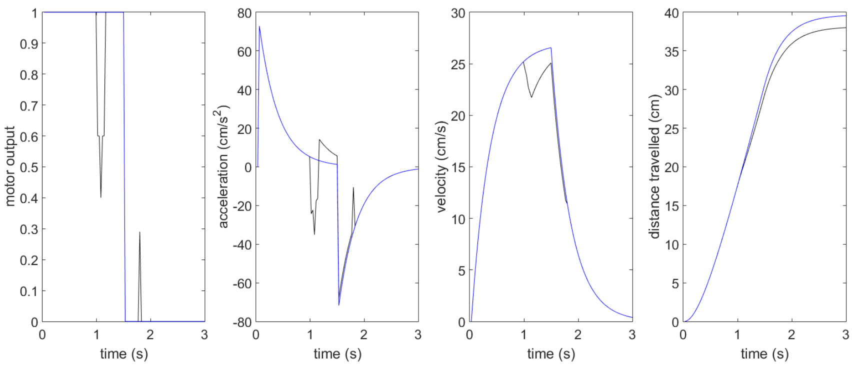

Figure S1: **Perturbation of an optimal input**. In this figure, we illustrate our proof with one particular example perturbation of one particular optimal trajectory. Kinematic values for the optimal trajectory are in blue, the same quantities for the suboptimal trajectory are in black. First panel: motor output. Second panel: acceleration. Third panel: Velocity. Fourth panel: distance traveled. Note that while the perturbed trajectory results in the desired velocity at the final time, the distance traveled remains insufficient to catch up with the other fish.

Velocity-dependent modulation of social forces is necessary for stable schooling

Fish schooling has been conceptualized as a decision process, where acceleration of the focal fish is determined by the relative position of the focal and partner fish and the current heading direction of the focal fish. On a one-dimensional track, the decision variable can be written as $d =$

$sign(v_{focal})(x_{partner} - x_{focal})$. The acceleration is then determined by $a = f(d)$, where f is the social force.

One necessary condition for schooling to be stable is for the trajectory of the focal fish to be stable even if the partner fish is stationary. Taking $x_{partner} = 0$ without loss of generality, we next consider a focal fish located at $x1$ (with $x1 < 0$) and moving with an initial velocity $v1 = 0$. Due to attractive social forces the focal fish will first accelerate towards its partner located at 0, leading to a decrease in their inter-agent distance but also an increase in the focal's velocity. Eventually, the focal fish will reach his partner at point 0, but in general it will still have some residual velocity at that point. After crossing the zero point, the focal fish will find that his velocity now starts to decrease even as his distance from the partner fish now starts to increase because inertia has not yet brought him to a stop.

We are interested in the value of the x coordinate of the focal fish at the moment his velocity reaches zero (let's call this value x_s). At this point, the social force map will invert because the sign of v_{focal} will invert and the cycle of acceleration and deceleration will begin anew. The long-term behavior of the focal fish will be stable only if $|x_s| < |x_1|$, at least for all values of x_1 larger than some limit x_L. If the opposite was the case, then each successive cycle of acceleration and deceleration would only take the focal fish further and further away from his partner leading to instability.

If social forces were not modulated by velocity, then, according to our equation of motion, $\int_{x1}^{x_s} f(x)dx = \int_0^{t_s} f(x(t))vdt = \int_0^{t_s} \frac{dv}{dt}vdt = \int_0^0 vdv$=0. We can break the integral $\int_{x1}^{x_s} f(x)dx$ into two parts, which measure the integral amplitude of the acceleration zone A_{ac} (between the values x1 and 0) $A_{ac}(x1) = |\int_{x1}^{0} f(x)dx|$ and the amplitude of the deceleration zone A_{dc} (between the values 0 and x_s) $A_{dc}(x_s) = \int_0^{x_s} f(x)dx$. For stability, we need to have $A_{ac}(x) < A_{dc}(x)$ at least for all values of x beyond a certain limit value x_L. If that were not true, then $\int_{-x}^{x} f(x)dx$ would be positive (for $x > x_L$), which would mean that each successive transition between points where the focal fish has velocity 0 requires a progressive increase in the distance between $x_s - x1$. Over time, this behavior would lead to catastrophic oscillations.

Measurements of the social force function in both mosquito-fish and golden shiners have shown that frontal acceleration zones have larger amplitudes than caudal deceleration zones, which, in isolation, would cause unstable schooling. However, measurements in these species have also shown that social forces are modulated by velocity. Velocity-dependent modulation of social forces can act as a damping factor and restore stability by dissipating excess motion energy similar to how damping can stabilize negative feedback control systems. Our mathematical argument demonstrates that velocity-dependence of social forces is a crucial precondition for stable schooling and

not an incidental feature of social interactions.
